# A rabbit osteochondral defect (OCD) model for evaluation of tissue engineered implants on their biosafety and efficacy in osteochondral repair

**DOI:** 10.3389/fbioe.2024.1352023

**Published:** 2024-05-03

**Authors:** Liangbin Zhou, Ki-Wai Kevin Ho, Lizhen Zheng, Jiankun Xu, Ziyi Chen, Xiangdong Ye, Li Zou, Ye Li, Liang Chang, Hongwei Shao, Xisheng Li, Jing Long, Yangyi Nie, Martin J. Stoddart, Yuxiao Lai, Ling Qin

**Affiliations:** ^1^ Musculoskeletal Research Laboratory of Department of Orthopaedics and Traumatology and Innovative Orthopaedic Biomaterials and Drug Translational Research Laboratory of Li Ka Shing Institute of Health Sciences, Faculty of Medicine, The Chinese University of Hong Kong, Shatin, Hong Kong SAR, China; ^2^ Department of Biomedical Engineering, Faculty of Engineering, The Chinese University of Hong Kong, Shatin, Hong Kong SAR, China; ^3^ Center for Neuromusculoskeletal Restorative Medicine, Hong Kong Science Park, NT, Hong Kong SAR, China; ^4^ Department of Biochemistry and Molecular Biology, Institute of Basic Medical Sciences, College of Basic Medicine, Hubei University of Medicine, Shiyan, China; ^5^ Zhongshan Institute of Changchun University of Science and Technology, Zhongshan, China; ^6^ Department of Chemical Pathology, Prince of Wales Hospital, The Chinese University of Hong Kong, Shatin, Hong Kong SAR, China; ^7^ Cardiovascular Research Institute, Icahn School of Medicine at Mount Sinai, New York, NY, United States; ^8^ Centre for Translational Medicine Research and Development, Shenzhen Institute of Advanced Technology, The Chinese Academy of Sciences, Shenzhen, China; ^9^ AO Research Institute Davos, Davos, Switzerland

**Keywords:** rabbit model, osteochondral defect, implant, tissue engineering, translational research

## Abstract

Osteochondral defect (OCD) is a common but challenging condition in orthopaedics that imposes huge socioeconomic burdens in our aging society. It is imperative to accelerate the R&D of regenerative scaffolds using osteochondral tissue engineering concepts. Yet, all innovative implant-based treatments require animal testing models to verify their feasibility, biosafety, and efficacy before proceeding to human trials. Rabbit models offer a more clinically relevant platform for studying OCD repair than smaller rodents, while being more cost-effective than large animal models. The core-decompression drilling technique to produce full-thickness distal medial femoral condyle defects in rabbits can mimic one of the trauma-relevant OCD models. This model is commonly used to evaluate the implant’s biosafety and efficacy of osteochondral dual-lineage regeneration. In this article, we initially indicate the methodology and describe a minimally-invasive surgical protocol in a step-wise manner to generate a standard and reproducible rabbit OCD for scaffold implantation. Besides, we provide a detailed procedure for sample collection, processing, and evaluation by a series of subsequent standardized biochemical, radiological, biomechanical, and histological assessments. In conclusion, the well-established, easy-handling, reproducible, and reliable rabbit OCD model will play a pivotal role in translational research of osteochondral tissue engineering.

## 1 Introduction

**FIGURE 1:**
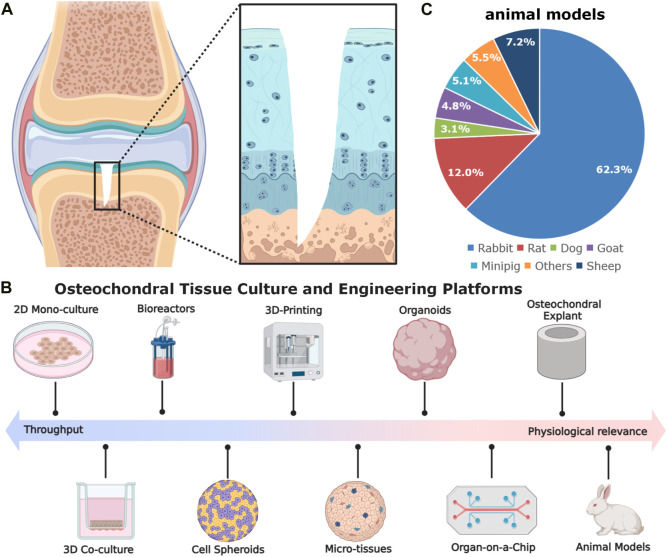
The schematic diagram of gradient osteochondral unit **(A)** and **(B,C)** *in vitro* and *in vivo* models used in osteochondral tissue engineering. The *in vitro* models comprise cell spheroids, micro-tissues, organoids, organ-on-a-chip, as well as explants. The commonly used animal models for OCD repair, inducing rabbit for 62.3%, rat for 12.0%, dog for 3.1%, goat for 4.8%, minipig for 5.1%, sheep for 7.2%, and others for 5.5% (González Vázquez et al., 2021) (Data was based on the papers published in 2010–2020).

Osteochondral defect (OCD) is a prevalent and debilitating orthopaedic disorder resulting from sports injuries, severe trauma, or physical diseases (i.e., osteoarthritis), triggering deformity, joint pain, and dysfunction (Zhou et al., 2020; Zengerink et al., 2006). The etiology of OCD is multifactorial and can vary depending on its location and severity (Chau et al., 2021; Bruns et al., 2018). It is defined as the focal areas of damage involving both the upper cartilage and a piece of underlying subchondral bone (Figure 1A) (Zhou et al., 2020). It affects millions of individuals globally and the most commonly affected joint is the knee, accounting for approximately 75% of all cases (Gaissmaier et al., 2006; Gorbachova et al., 2018; Chau et al., 2021). The incidence of OCDs in the general population is unknown, but it is estimated that they account for 5%–15% of all knee injuries (Gaissmaier et al., 2006; Chau et al., 2021). The prevalence of OCD increases with age, as well as with certain risk factors such as obesity, physical activity, and previous trauma (Bruns et al., 2018). For OCD repair and regeneration, to date, a series of clinical therapeutic options (nonsurgical or surgical treatments) including physical therapy, oral and topical medications, injections, platelet-rich plasma and stem cell treatments, arthroscopic debridement, chondroplasty, mosaicplasty, osteoarticular transfer system, microfracture, autologous chondrocyte implantation (ACI), matrix-induced ACI (MACI) are accessible (Zhou et al., 2020; Howell et al., 2021). Although the above-mentioned treatments have brought some exciting news to some degree, none of them have shown a complete and consistent functional repair of OCD with long-lasting hyaline cartilage. Therefore, more advanced and valid tissue-engineered therapeutics are highly desirable for fully and durably repairing osteochondral injuries, especially in terms of osteochondral interface (Yildirim et al., 2023).

In osteochondral tissue engineering, relevant disease models are a crucial step for evaluating the implant’s biosafety and efficacy of cartilage and subchondral bone regeneration and can bridge some preclinical scientific discoveries to ultimate clinical applications (Meng et al., 2020; Vainieri et al., 2020; Yang et al., 2023). Apart from some *in vitro* and *ex vivo* models (e.g., cell culture methods, organoids, organ-on-a-chip, and osteochondral explants), small animal models (e.g., mice, rats, and rabbits) together with large animal models (e.g., sheep, goats, dogs, horses, monkeys, and emu) have been most widely utilized for evaluating different strategies for OCD healing (Cook et al., 2014; Meng et al., 2020) (Figure 1B). Each of them has pros and cons. For rabbits, there are some controversies of using the rabbit model in OCD repair. About 20 years ago, using rabbits for cartilage repair studies was not regarded as useful. OCD repair in rabbits was less challenging as compared with that in large animals or patients, probably due to the higher metabolic activity, lower body weight, different loading conditions, faster bone growth rate, and higher regenerative capacity (Chu et al., 2010; Moran et al., 2016; Meng et al., 2020). Moreover, the reported studies in the literature used younger rabbits (skeletally immature) and the observation window was also rather short and mostly did not exceed 12 weeks. However, they have shown some advantages in the following aspects: 1) suitable joint size: rabbits have larger joints compared to rodent models, making them more suitable for assessing cartilage repair; 2) easier surgical procedures: the size of rabbits allows for easier surgical procedures and specimen handling; 3) rabbits have a cartilage thickness ranging from 0.25 mm to 0.75 mm, which is thicker than mice and rats (Cook et al., 2014; Meng et al., 2020); 4) more cost-effective, manageable, easy to accommodate, and available in large-scale genetically homogenous groups compared to large animal models; 5) faster bone turnover rate than larger animals, which can reduce the duration of the experiments. These merits make rabbit models to be effective alternatives (roughly accounting for 62.3% among animal models) for studying osteochondral repair in orthopaedic research (Figure 1C).

Here, a standard rabbit OCD model is established by a minimally invasive surgical method, which is described in detail with important steps. Several assessment approaches are presented to evaluate the extent of osteochondral repair. The abovementioned model used for evaluating the implant’s *in vivo* biosafety and efficacy in osteochondral tissue engineering will contribute greatly to the R&D of innovations towards clinical applications.

## 2 Overview of the procedure

The procedure consists of two parts: 1) the establishment of the rabbit OCD model for scaffold implantation, and 2) the evaluation and analysis methodologies. More specifically, at the outset, the skin, fascia, and joint capsule were incised sequentially on the medial aspect of the knee. Then the subluxation of patellar tendon as well as the bending of knee were performed to visualize the hyaline cartilage. Through the core-decompression drilling, a focal defect with a predetermined size was created on medial femoral condyle and after scaffold implantation, the knee extension and reduction of the patella dislocation were accomplished before wound closure. At each observational time point, the blood samples were collected for hematology tests and pain evaluation was performed. At the experimental end time points, rabbits were sacrificed for sample harvest. The isolated major organs could be assessed by H&E staining to confirm the scaffolds’ *in vivo* biosafety. The collected femoral condyles were evaluated by various methods, including high-frequency (HF) ultrasound, biochemical assays, micro-computed tomography (micro-CT), histology, immunohistochemistry (IHC), and mechanical testing.

## 3 Materials and methods

### 3.1 Experimental design

#### 3.1.1 Implantation biomaterials

**FIGURE 2:**
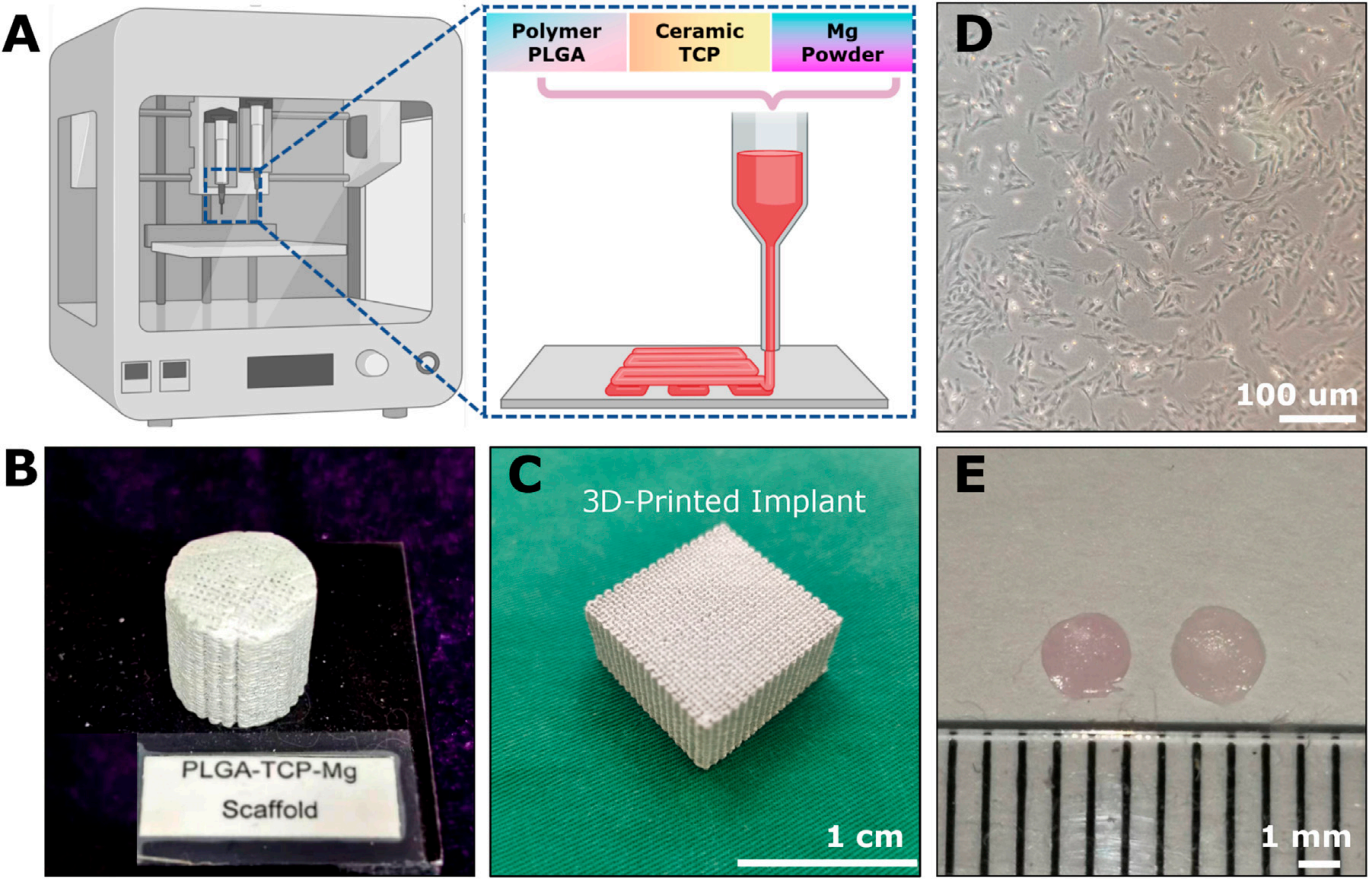
The example of implantable scaffolds and cell sources for osteochondral repair. **(A)** The 3D-printing platform for fabricating Mg-based scaffolds through polymer PLGA, ceramic TCP, and Mg powders. The cylindrical **(B)** and cubic **(C)** appearance of the 3D-printed Mg-based porous bioactive composite implants. **(D)** The third passage of Rabbit BMSCs. **(E)** 2-weeks-cultured hyaline-like disc-shaped tissue-engineered chondrocyte pellet (TeCP).

**FIGURE 3:**
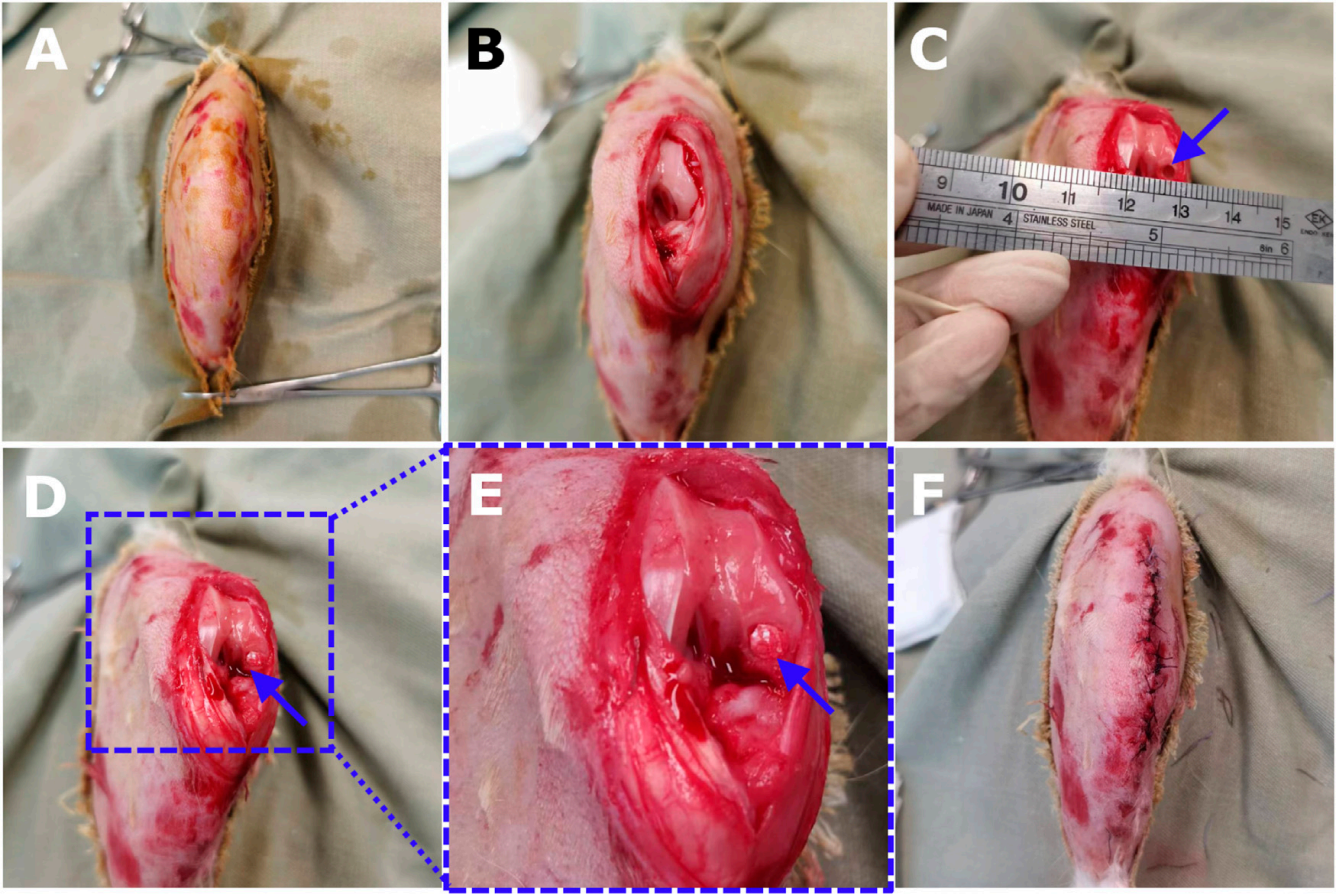
The surgical procedures of core decompression drilling for creating OCD in a rabbit model and the scaf-fold implantation. **(A)** After analgesia and anesthesia, preoperative shaving was properly conducted for the right knee joint of an 18-week-old New Zealand rabbit. **(B)** The knee was opened through a medial parapatellar longitu-dinal incision. The patella was dislocated laterally to expose the femoral condyle and the knee was maximally flexed. **(C)** Using the grafting instrument to create a cylindrical OCD (3 mm × 3 mm) on the weight-bearing area of the medial femoral condyle. **(D)** Scaffold implantation by gently pressing the construct into the defect bottom. **(E)** The magnification showed the dill site and the tailored implant fitted well with the defect. **(F)** The patella was relocated, and the joint was mobilized in a full range of motion to guarantee no dislocation of the bioimplants. Then the joint capsule, fascia, and skin were sewed layer by layer.

In the scenario of osteochondral repair, the biomaterial scaffolds could provide three advantages in creating supportive microenvironments, forming advanced drug delivery systems, and offering extra mechanical support (Zhou et al., 2020; Zhou et al., 2023). At present, various biomaterials have been developed and evaluated for OCD repair, such as microsphere, fibrous, porous, metal, decellularized matrix, hydrogel, and composite scaffolds (Erisken et al., 2008; Erisken et al., 2011; Zhou et al., 2020; Lesage et al., 2022; Zhou et al., 2022). Each type has its pros and cons in terms of biocompatibility, degradability, porosity, and mechanical strength (Zhou et al., 2022). Bone and cartilage have distinct compositions, structures and functions, so they require different types of biomaterials for biomimetic scaffold fabrication. Accordingly, bi-layered, tri-layered, and gradient scaffolds-based strategies are proposed to mimic the intrinsic gradient properties of the osteochondral junction (Zhou et al., 2020; Lesage et al., 2022). Here, we fabricated a novel Mg-based scaffold via low-temperature rapid prototyping technology (Figures 2A–C) for subsequent implantation and evaluation. The 3D-printed porous pre-shaped Mg-based bioactive scaffolds (cylinder: 3 mm in diameter and 3 mm in height; Figure 3E) were implanted into the rabbit OCDs.

#### 3.1.2 Cell sources or/and biochemical cues

The cell sources for osteochondral repair can be divided into two categories: endogenous cells and *in vitro*-manipulated cells no matter if they are autologous or allogeneic (Ng et al., 2016; Yang et al., 2020). Typically, these *in vitro*-manipulated cell sources range from tissue-specific cells (e.g., chondrocytes and osteocytes) (Davies and Kuiper, 2019; Cao et al., 2020) to progenitor cells (e.g., BMSCs, ADSCs, articular cartilage progenitor cells (ACPCs), synovial membrane-derived MSCs (S-MSCs) and iPSCs) (Yang et al., 2020; Zhou et al., 2023). Typically, BMSC- and chondrocyte-based engraftments are widely used for OCD healing (Figures 2D,E). Besides, many exogenous biochemical cues such as chemokines, cytokines, growth factors, small molecule drugs, peptides, mineral ions, gene-targeting factors, extracellular vesicles, and some immunomodulatory and anti-inflammatory agents have been utilized for chondral or OCD regeneration (Zhou et al., 2023).

#### 3.1.3 Study duration

Several implantation time courses have been selected for *in vivo* investigation. However, there is no recommended optimal duration of the study. The consideration for selecting study endpoints usually relies on the study aim, animal numbers, grouping, housing space, and feeding prices. They have been reported to range from 2 to 48 weeks (Harada et al., 2015; Kazemnejad et al., 2016; Chen et al., 2018; Jia et al., 2018; Duan et al., 2019; Xing et al., 2021). However, the most frequent ones are between 4 and 12 weeks, which can reflect the short-to mid-term osteochondral healing respectively (Harada et al., 2015; Xing et al., 2021). Long-term observations (e.g., over 1 year) are also essential for checking whether the approaches would finally result in fibrotic condition. Here, we selected 12 weeks as the study endpoints for analyzing PTM scaffold implantation.

#### 3.1.4 Defect model

Approximately 73% of animal studies utilized acute defect models, while 24% of studies evaluated regeneration in chronic defects despite chronic models being more representative of human defects (González Vázquez et al., 2021). Based on the financial and ethical concerns, we focus in this paper on induction of an acute trauma-relevant OCD model by drilling a standard full-thickness femoral condyle defect in rabbits. For defect shape, irregular-shaped defects (e.g., rectangle and square) are infrequently used (Kondo et al., 2019). The most popular ones are the cylindrical defects on the trochlear groove or the load-bearing area of the medial femoral condyle (Zhu et al., 2020; Steele et al., 2022). To the best of our knowledge, the latter location can better mimic the clinical traumatic OCDs due to the harsh mechanical environments with the joint. Meanwhile, the bilaterally created defects can minimize the rabbit numbers for *in vivo* study. The size of defects varies from 2–5 mm in diameter, and 1–5 mm in depth (Meng et al., 2020), which are mainly determined by the skeletal maturity. Older rabbits have wider femoral condyle surfaces and can accommodate larger defects. Besides, drilling a too-deep defect should be avoided to prevent the leak of bone marrow, unless the designed procedure requires the subchondral bone to be microfractured.

#### 3.1.5 Control group

The control group could be set as the empty defects (namely, spontaneous healing), native healthy osteochondral tissues of the contralateral knee, and defects repaired by some currently clinical techniques (e.g., microfracture and ACI). The product type and associated regulatory requirements determined the selection of the comparator group in clinical or preclinical trials (González Vázquez et al., 2021). Here, we selected the untreated empty defects as the negative control group.

### 3.2 Required materials

#### 3.2.1 Animal species and selection

Due to the suitability for various experimental procedures, orthopaedic research often employs rabbits as a small or medium-size animal model, especially for the assessment of cartilage repair. So far, there is no consensus on the specific age of skeletally mature rabbits with a range from four to 9 months old. This protocol used 4-month-old (relatively young rabbits and their growth plates were not fully closed) New Zealand White (NZW) rabbits due to fewer health problems and favorable behavioral characteristics of this strain. Younger rabbits of 9 weeks of age have also been applied as models for OCD regeneration, but they exhibit faster rates of skeletal alteration and turnover in both cartilage and bone (Yan et al., 2015; Meng et al., 2020). Considering sex-related differences in the modulation of osteogenic and chondrogenic processes, cartilage thickness, and bone density (Irie et al., 2005; Meng et al., 2020), it is essential to include both sexes for *in vivo* studies. Each study designer should conduct a power analysis to determine an adequate sample size to address study objectives. Using authors’ institution as an example, before being transferred to our animal house, these rabbits underwent a disease screening protocol that involved gross examination, infectious agent testing, parasitology, microbiology, and serology. They were allowed a 72-h acclimation period before surgery. They were housed in a barrier facility free of parasite and virus and exposed to a 12-h light/dark cycle. The humidity and room temperature were kept at 35%–66% and 20°C respectively. They had daily access to food and water *ad libitum*. All animal procedures depicted in this protocol strictly followed the license to conduct animal experiments approved by the Animal Experimental Ethics Committee (AEEC) of CUHK (Ref No. 17-172-AOF). In this study, a total of 14 rabbits were used, including 12 rabbits for the surgical creation of OCD (*n* = 6 for empty Ctrl group, and *n* = 6 for the implantation of example scaffolds) and two rabbits without any surgeries (one 6-weeks-old NZW rabbit and one 18-weeks-old NZW rabbit for verifying the detection capability of HF-ultrasound).

#### 3.2.2 Personnel and expertise

Four staff including two primary surgeons, one veterinary technician, and one assistant technician are highly recommended to work as a team to conduct this surgical protocol. Dental or medical surgery training is required for the two surgeons. All the theoretical and practical skills should be possessed by the veterinary technician for experimental animal studies. Besides, the assistant technician could provide extra supplies. Rational professional training-based examination and endorsement are required for all personnel involved.

#### 3.2.3 Equipment and tools

1. Low-temperature rapid-prototyping machine (CLRF-2000-II, SUNP BIOTECH).
2. Freeze dryer (Bo Yi Kang FD-1-50, China).
3. Vacuum pump (VLP120-230, Thermo Fisher).
4. Autoclave (Tomy SX-500).
5. High-frequency (HF) ultrasound imaging system (VevoLAZR, VisualSonics, Inc.).
6. Linear array transducer (MS700, VisualSonics, Inc., Toronto, Canada).
7. uCT40 (Scanco Medical AG, Brüttisellen, Switzerland).
8. Automated rotary microtome (Leica RM2165, Leica, Germany).
9. Water batch for paraffin sections (Hl1210, Leica).
10. Flattening table for clinical histopathology (Hl1210, Leica).
11. Microscope (Leica DMLS).
12. Microscope (Leica DM5000B).
13. Uniaxial mechanical testing machine (H25KM, Hounsfield/Tinius Olsen).
14. Dynamic ultra-micro hardness tester (DUH-211, Shimadzu).
15. Portable small animal anesthesia machine (R530, RWD).
16. Veterinary anesthesia ventilator (3002PRO, midmark).
17. Heated surgical table (Short-Line).
18. Surgical lamp (PentaLED 12, Rimsa).
19. 1029-XL Incapacitance Tester (Columbus, Ohio, US).
20. Diamond band saw (EXAKT 300CP, EXAKT Technologies, Inc.).
21. Saw microtome (SP1600, Leica).
22. Polycut sectioning system (SM2500E, Leica).
23. StepOne Plus 96 qPCR system (Applied Biosystems).
24. Refrigerated centrifuge (5424 R, Eppendorf).
25. Refrigerated centrifuge (LMC-4200R, Biosan).
26. Protein electrophoresis and blotting (Bio-Rad).
27. Electric razor (Panasonic).
28. Electronic calipers (Qfun).
29. Handheld dental drill (YuTong).
30. Adson toothed forceps (Instrapac^®^, Vernacare).
31. Curved mosquito forceps (YIJIANMEI).
32. Double-ended retractor (Richardson-Eastman).
33. Suture scissors (Cofoe).
34. Needle drivers (ARTMAN INSTRUMENTS).
35. 1.5 · 70 mm/2.0 70 mm/3.0 70 mm stainless steel drill bit (AYMS).
36. Bone rongeur (FO412R, LUER).

#### 3.2.4 Reagents and surgical materials

1. E-Z scrub™ brush (BD Products).
2. Sterile head cover, shoe covers, gloves, gown, and facemask (CENDY).
3. Suction canister, line, and handle (Patterson Veterinary).
4. Sterile disposable scalpels (BAISHUN PET).
5. Chlorhexidine solution (Kahuvet).
6. Absorbent underpads (Thermo Fisher).
7. Surgical drapes (Pusner lucewin).
8. Surgical towels (ULTECHNOVO).
9. Nonwoven gauze sponges (CENDY).
10. A 22G · 1† intravenous catheter (Medigrative).
11. Winged infusion set (YIKANG).
12. Povidone iodine (ANNJET).
13. Sterile 1 mL/3 mL/5 mL/50 mL syringes (Thermo Fisher).
14. 16G 1† and 23G 1† needles (Thermo Fisher).
15. 5–0 VICRYL PLUS sutures (VCP303H-Ethicon).
16. Tissue adhesive (3M Vetbond).
17. Bandaging tape (Cofoe).
18. Surgical ruler (AROSurgical ™) and vernier caliper (BiaoKang SL01-1).
19. Ethanol, ≥99.5% (Sigma-Aldrich).
20. Ketamine and Xylazine (Alfasan).
21. Buprenorphine (Patterson Veterinary).
22. Pentobarbital (25%, Euthasol).
23. Amoxicillin (Centrafarm).
24. Physiological saline solution (Pharmex).
25. Tissue specimen containers (Thermo Fisher).
26. 10% neutral-buffered formalin (Sigma-Aldrich).
27. EDTA (ethylenediaminetetraacetic acid) solution (Thermo Fisher).
28. Paraffin (Leica Biosystems).

#### 3.2.5 3D-printing fabrication of example implants

Here, we used the Mg-based biomaterials as example scaffolds. The 3D-printing of Mg-based scaffolds was performed via low-temperature rapid prototyping technology according to a previously well-established protocol (Figure 2A) (Lai et al., 2019). Briefly, all the chemicals used were analytical research grade. The PLGA polymers (polylactide: polyglycolide = 75%: 25% with an MW of 100,000 and a viscosity of 1.6 dL/g), tricalcium phosphate (TCP) powders, and Mg particles (50–80 um) were dissolved in 1,4-dioxane as homogeneous solutions to form PTM scaffolds (ratio of PLGA/TCP/Mg: 16/2/2 wt%). Then a uniform liquid paste was formed by stirring the mixture vigorously overnight. By using an advanced low-temperature rapid-prototyping machine, Mg-based porous composite scaffolds were printed at −30°C. Lastly, these fabricated porous PTM scaffolds (Figures 2B,C) with various predesigned parameters were lyophilized (20–40 Pa, 24 h), sterilized (γ radiation), and stored in sterilized packages for subsequent investigations.

### 3.3 Experimental protocol

#### 3.3.1 Rabbit fur removal (optional, 2–3 days before surgery)

1. To minimize fur and save time, rabbits can optionally be shaved 2–3 days before surgery. Before fur removal, rabbits need to be weighed to calculate the drug dosages for anesthesia.
2. To restrain the rabbit, the rabbit’s scruff was firmly grasped and its head was placed under the handler’s upper arm, while its hindquarters and back were supported by the forearm.
3. Rabbits were anesthetized by a mixture of 50 mg/kg body weight ketamine (l00 mg/ml) and l0 mg/kg body weight xylazine (20 mg/ml) in a single syringe with 23 G needle intramuscularly. The level of anesthesia will be examined by the hind toes, regular respiratory rate, and skin.
4. We removed the rabbit’s fur with surgical clippers after confirming that the rabbit was fully sedated.
5. As an indicator of anesthesia recovery, the vital signs (e.g., oxygen saturation, pulse rate) of rabbit (maintaining a sternal position) should be monitored.

#### 3.3.2 Preoperative preparation

6. Autoclave the drilling equipment and surgical tool set.
7. Prepare enough PTM scaffold packages for the following implantation.
8. Prepare the surgical table by setting the warm-temperature mode and covering it with an absorbent underpads.
9. Set up the handheld dental drilling apparatus and surgical tool sets. Locate them on an adjacent non-operating table, ensuring that they are covered by two sterilized surgical drapes respectively.

#### 3.3.3 Surgical induction

10. Before surgical operations, the rabbit was administered pre-emptive analgesic drugs intramuscularly, for example, Temgesic (buprenorphine, 0.02–0.05 mg/kg) and anesthetized intramuscularly with a mixture of ketamine (50 mg/kg) and xylazine (10 mg/kg).
11. The surgical area was shaved with an electric shaver (Figure 3A). Next, scrub the surgical sites with a 0.5% solution of chlorhexidine and sterilize them with iodine (100 mg/mL).
12. The rabbit was fixed in the supine position on the surgical table.
13. A winged infusion set for intravascular (i.v.) administration was inserted in the rabbit’s ear. The anesthesia was maintained by 2.5% Pentobarbital i.v. Infusion if the rabbit showed any signs of arousal or discomfort.
14. The non-surgical sites of the rabbit were covered by sterilized drapes.

#### 3.3.4 Defect generation and implantation

15. Here, we only select the right hind leg for defect generation and implantation. The contralateral knee remained empty to serve as untreated control. Some studies used both legs simultaneously, but it would lead to difficulties in locomotion, drinking, and eating of rabbits, especially within 1-week post-operation.
16. Bend the knee slightly (with the bending angle of *20%–30%) and identify the patellar tendon by palpation. Then mark the incision site at *5 mm medial to the tendon center.
17. A longitudinal (parallel to the tendon) incision was made on the skin layer.
  (1) Fascia, underlying joint capsule (pink color) and small blood vessels are now visible.
  (2) Press gauze on the hemorrhages gently if you hurt any blood vessels until the bleeding stops.
  (3) If you hit a bigger blood vessel and it keeps bleeding even after you press on it with gauze, use curved mosquito forceps to clamp the area lightly and stitch it up if needed to stop bleeding.
18. Incise the same spot deeper to reach the fascia (thin and translucent) and the joint capsule (thicker and opaque) successively. As soon as the scalpel reaches the lower border of the femoral condyle and the upper border of the tibial head, terminating the incision process immediately is needed to avoid damaging the bone.
19. To visualize the medial femoral condyle, the joint was maximally extended, and the patella was dislocated laterally. Then flex the joint with the bending angle of *75% and maintain the dislocation of patella till the completion of scaffold implantation.
20. Adjust the angle of joint flexion as well as the joint stability and slightly more incision with the joint capsule if needed for easily accessing the medial femoral condyle (Figure 3B).
21. The surrounding tissues were retracted via retractors and forceps to thoroughly expose the drilling site.
22. A small hole in the middle of the plateau area (load-bearing site) of the medial femoral condyle was made by the 16G needle.
23. Use the drill bit (1.5 mm in diameter) to drill at the entry site and ensure no slippage. End the drilling until it reaches 1 mm in depth.
24. Use the sterilized physiological saline solution (4°C) to rinse the entry site during the whole drilling process in order to rinse debris and avoid local overheating.
25. To avoid the collapse of subchondral bone, using the core-decompression technique with a drill bit (3.0 mm in diameter) to drill at the aforementioned 1.5 mm × 1.0 mm hole to generate a defect with 3 mm in diameter and 3 mm in depth (Figure 3C), and also rinse the site and ensure no slippage throughout the whole process. Using the surgical ruler and vernier caliper to validate whether the diameter and depth of the defects were exceeding the range of 3 ± 0.2 mm, ensuring the precision of the generated defects.
26. Debris from the interior and edges of the cylindrical OCD were removed and the bottom was flattened. Then use a gauze to dry the defect.
27. Implant the biomaterial scaffold or tissue-engineered graft into the cylinder defect (Figures 3D–E). Meanwhile, the defects without any interventions and implantations were considered as the empty defect controls.
28. The patella was relocated and the full range of motion of the knee joint was restored to guarantee no dislocation of the implants.
29. Be careful of sterile operation during the surgery to avoid infection or even death of rabbits.

#### 3.3.5 Wound closure

30. Here, we applied 5–0 VICRYL PLUS sutures for wound closure. The joint capsule was closed in a discontinuous pattern with the assistance of scissors, toothed forceps, and needle drivers.
31. For the fascia layer, it was sutured a continuous pattern.
32. For the skin, it was sutured in an intracutaneous interrupted pattern. To ensure closure of the outer skin layer, it was essential to place additional stitches in the wound center (Figure 3F).
33. Hemorrhage was controlled. The wound area was cleaned with sterilized gauze and disinfected with iodine (100 mg/mL), and a tissue adhesive was used if necessary.

#### 3.3.6 Postoperative care

34. Pro phylactic Amoxicillin (20 mg/kg; Centrafarm) was administered by i.v. Infusion as a preventive measure.
35. For postoperative analgesia, then 0.02–0.05 mg/kg body weight buprenorphine hydrochloride (Temgesic^®^, Reckitt Benckiser Healthcare Ltd., 28 Hull, East Yorkshire, United Kingdom) was injected twice daily on the first day and intramuscularly for 3 consecutive days post-operation to minimize the pain.
36. During the recovery period, a heat source (a lamp for warming the animals) will be provided to the rabbits. The rabbits were transferred to the animal facility when they had independent breathing and stable saturation, and they were monitored individually.
37. Rabbits were allowed free cage activity soon after the operation and enough water and food. Check animals at least twice daily within 1 week postoperatively and thereafter once daily until sacrificing them.
38. Record the health conditions of rabbits. If they show any sign of illness or disability that violates the animal welfare check list (e.g., wound infection, lack of appetite, and difficulty walking), report to the veterinarian for help.
39. At every indicated time point, collect 2–3 mL serum samples via ear arteries. After collection of the whole blood, allow the blood to clot (15–30 min) by leaving it undisturbed at room temperature. Remove the clot by centrifuging at 1,000–2,000 × g for 10 min in a refrigerated centrifuge.
40. Immediately transfer the supernatant into a clean polypropylene tube. Then the apportioned 0.5 mL aliquots of samples should be stored at −20°C or lower for the following analysis.

#### 3.3.7 Euthanasia and sample collection

**FIGURE 4:**
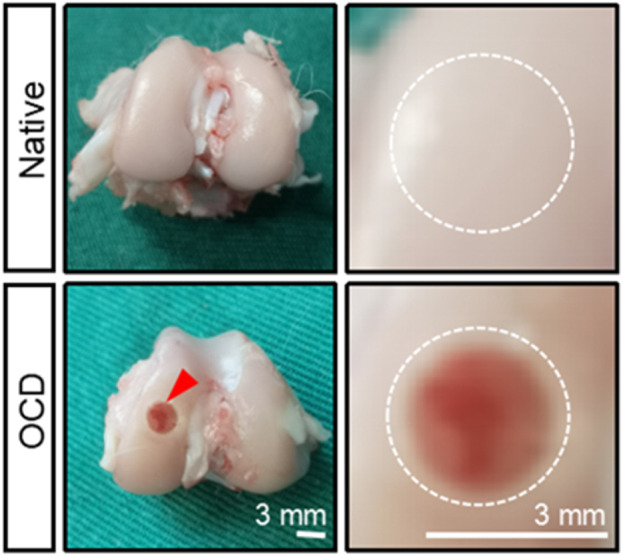
The gross appearance of native healthy cartilage and an OCD in a rabbit model. The defect was visually seen, and its diameter and depth were confirmed by a caliper. The bottom of the defect was flattened with a drill bit, and the defect was rinsed with saline when and after drilling to cool down and remove some tissue debris.

41. At several endpoints (e.g., 4 weeks, 8 weeks, and 12 weeks post-operation), the rabbits were weighed to verify their weight if they were within the normal range and calculate drug dosages.
42. A mixture of ketamine/xylazine (50 mg/kg and l0 mg/kg; Alfasan) in a single syringe with 23 G needle was administered intramuscularly.
43. Rabbits were euthanized by intracardial (i.c) injection of excessive pentobarbital (25%, Euthasol).
44. The verification of euthanasia was by the lack of cardiovascular function, such as respiration, heartbeat, and eye reflex.
45. Three rabbits were randomly selected from each group at each pre-designed time point after the surgery. Rabbits could be placed in a restraint device for blood collection via 25G needles or butterfly catheters on the site of the marginal ear vein (1–3 mL) at each pre-designed time point. Rabbit blood was collected in a yellow evacuated blood collection tube (containing clot activator to promote clotting). Allow the blood to clot at room temperature for 15–30 min before getting to the next step to centrifuge the tube for 10 min at 2,000 to 2,500 × g. The serum would be the clear yellow liquid above the clot. Then the tube was carefully removed from the centrifuge and the desired serum component was transferred to a clean tube before storing it at −20°C or lower for the following analysis.46. To collect the joint sample, surgical cuts were performed on the left hind joint by utilizing scissors and a scalpel to expose the synovial membrane and distal femur. The complete distal femur was removed with a circular saw and shown in Figure 4.
47. The specimen was cleaned of any remaining irrelevant tissues and reduced to a smaller dimension by applying a diamond saw.
48. Then to collect major organs (e.g., heat, liver, kidney, lung, spleen), spray the rabbit with 70% ethanol and place it on its back on a dissection board. Pin the limbs to secure the rabbit in position.
49. Make a midline incision from the lower abdomen to the neck with a scalpel. Cut through the skin and muscle layers but avoid damaging the underlying organs. Then collect the heat, liver, kidney, lung, and spleen samples.
50. The collected specimens, including distal femurs and major organs, were placed in separate 50 mL Falcon tubes containing 4% paraformaldehyde solution (40–50 mL) on a shaker for 48–72 h at 25°C.

#### 3.3.8 Subsequent analysis

This rabbit OCD model has been used several times in osteochondral tissue engineering (Cheuk, 2009; Cheuk et al., 2011; Guo et al., 2020; Meng et al., 2020; Monteiro et al., 2022). It could help to examine the dual-lineage osteochondral regeneration capability as well as *in vivo* biosafety of grafting scaffolds and tissue-engineered products. The following sections would discuss an example of such a study and the data obtained from it.

**FIGURE 5:**
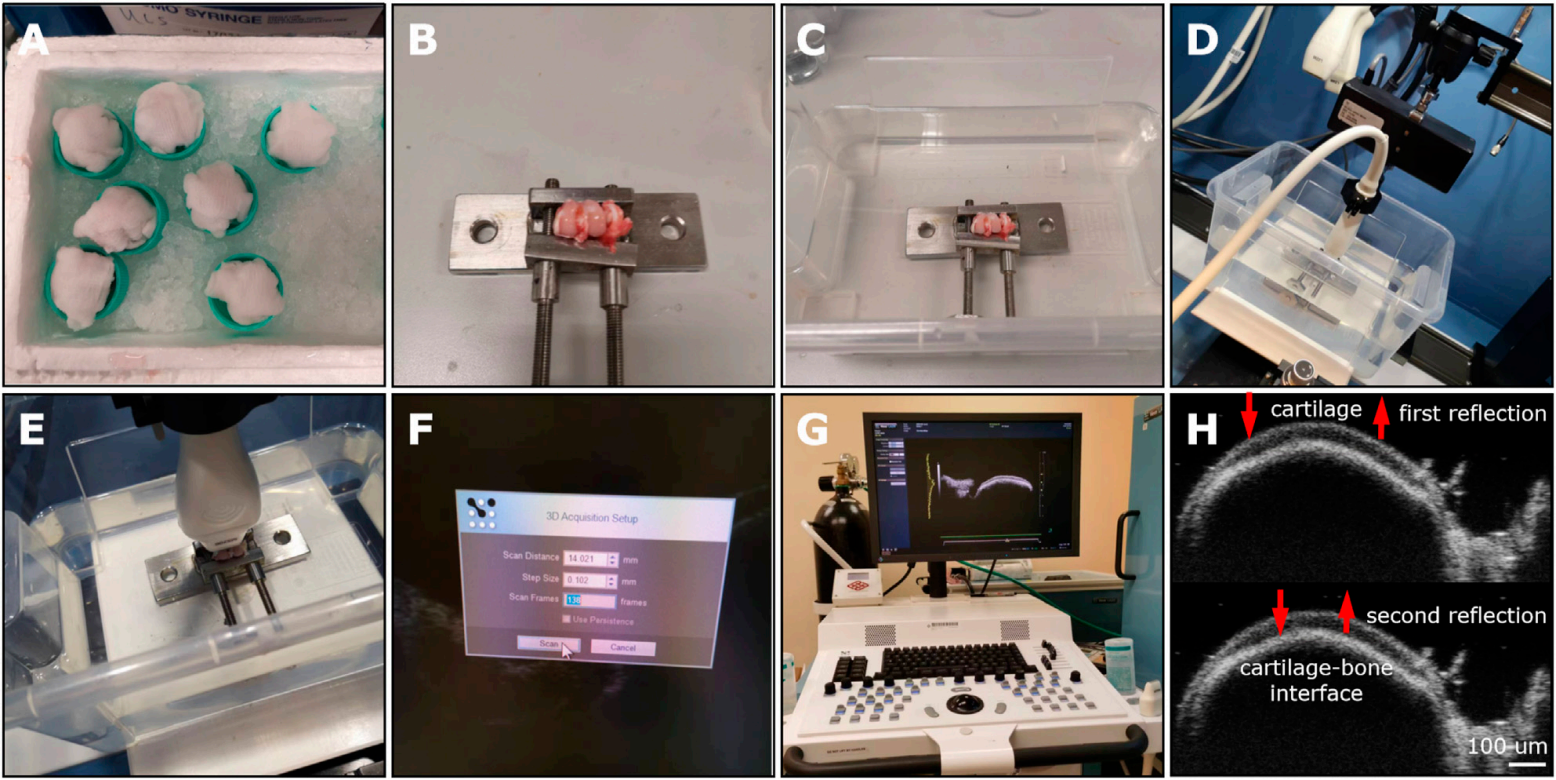
The HF-ultrasound imaging platform for the preliminary evaluation of OCD repair in a rabbit model. **(A)** The stored femur samples from −80°C were covered and thawed by medial gauze soaked with physiologic saline solution for a minimum of 0.5 h at 25°C. **(B)** The rabbit femur was immobilized by an adjustable immobilization equipment to place the defect area parallel to the horizontal line. **(C)** The immobilized femoral condyle was located at the container bottom and soaked in 0.9% NaCl solution. **(D)** Select the proper transducer (MS 700). **(E)** Adjust the transducer perpendicular to the defect area. **(F)** Select proper scanning parameters, including 14.021 mm for scan distance, 0.102 mm for step size, and 138 frames. **(G)** The HF-ultrasound imaging platform. **(H)** Two reflected HF-ultrasound signals were collected for clearly distinguishing the cartilage surface and the bone-cartilage interface.

**FIGURE 6:**
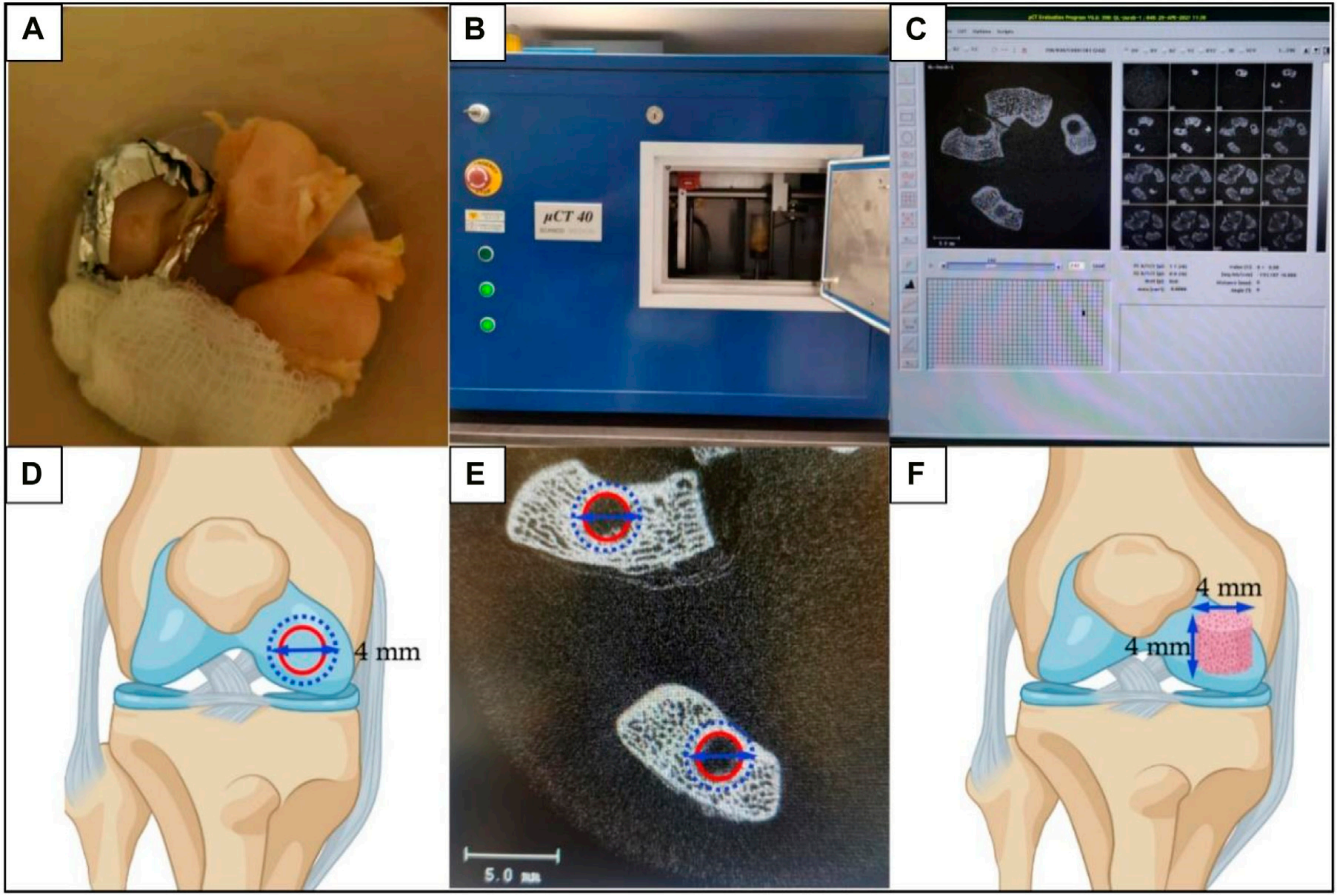
The micro-CT scanning and subsequent morphometrics analysis for subchondral bone assessment. **(A)** Trimmed femur samples were placed in a sample holding tube and soaked with 0.9% NaCl solution. Using gauze and foam boards to adjust the femurs and place the defect area parallel to the tube bottom. **(B)** The sample holding tube was placed into the CT machine for scanning. **(C)** Scanned images were reconstructed. **(D)** Schematic diagram of the defined ROI as a disc (4 mm in diameter) to contain the previously created OCD (3 mm in diameter). The outer blue dashed circle represented the defined ROI and the inner red circle represented the OCD. **(E)** The ROI was thresholded to reflect mineralized bone. **(F)** Images were morphed to create the VOI which was defined as a 4 mm × 4 mm cylinder (perpendicular to the defect surface) to evaluate the regenerated subchondral bone.

**FIGURE 7:**
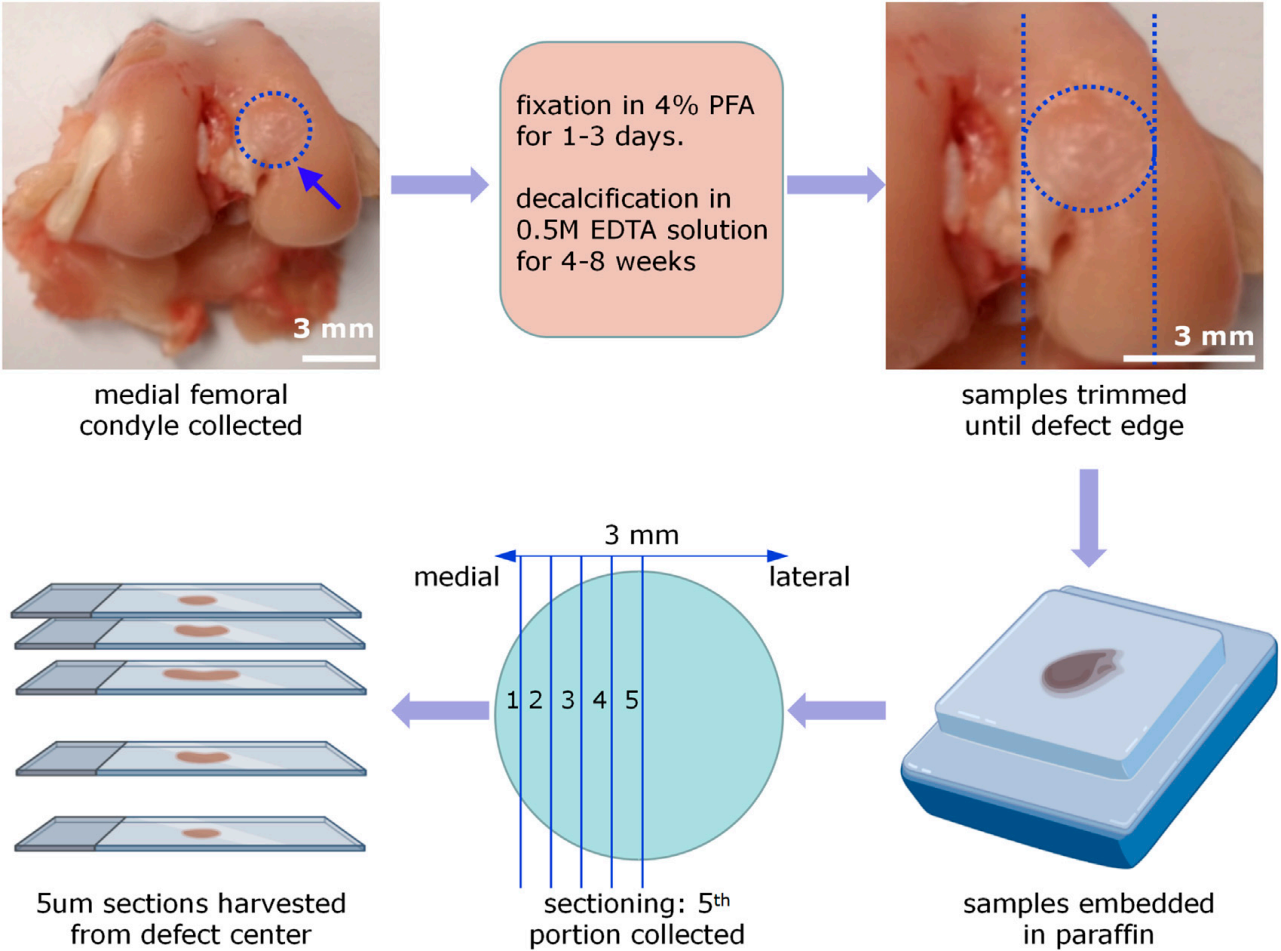
Procedures of rabbit femur sample processing and sectioning. The medial femoral condyles were collected, fixed by 4% PFA solution for 1–3 days, and decalcified by 0.5M EDTA solution for 4–8 weeks. Then trim the samples until 1 cm away from the defect edge. After embedding samples by paraffin, an OCD was virtually divided into 10 parts. Five um paraffin sections were collected in every other portion. Only sections from the fifth portion were selected for the following histology and histomorphometry analysis.

**FIGURE 8:**
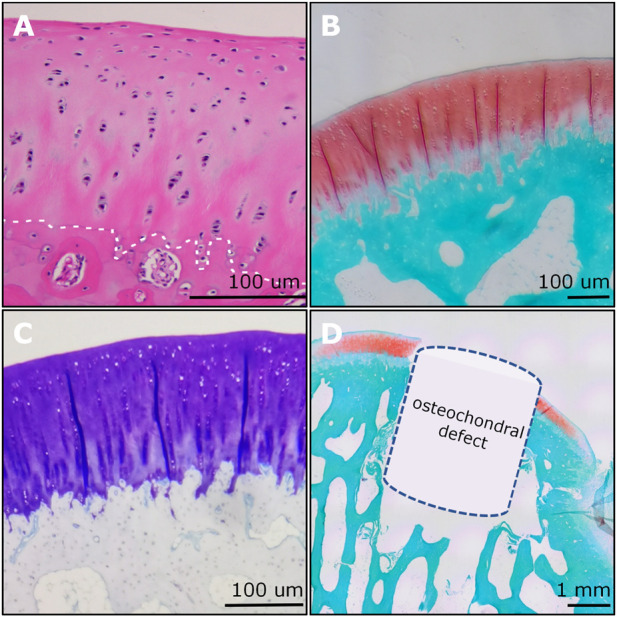
Examples of histological imaging analysis of rabbit osteochondral junction. **(A)** The H&E staining showed the gradient structure of the osteochondral unit. The chondrocyte nucleus was stained blue to dark. The white dashed line indicated the tidemark. **(B)** The SO&FG staining revealed the proteoglycan contents in cartilage. **(C)** The T&B staining indicated the GAGs in cartilage. **(D)** An OCD (3 mm × 3 mm) was visualized and verified via SO&FG staining. **(A)** and **(D)** were reproduced with permission from the publications (Zhou et al., 2022; Zhou et al., 2023).

51. Perform hematology tests of serum samples to check the implant’s *in vivo* biosafety. Before analyzing the rabbit serum samples, the DXC 800 automated biochemical analyzer (Beckman Coulter, United States) shall be calibrated and validated. Then explore the blood biochemistry following the manufacturer’s instructions and quality control procedures, and record and interpret the results of liver function indicators (e.g., ALB, ALT, GLB, AST) and kidney function indicators (e.g., serum Cre and serum BUN) according to reference range of various indicators in rabbits (Washington and Van Hoosier, 2012). Lastly, the used blood materials and related experimental consumables must be disposed of according to the biosafety guidelines.
52. Perform pain evaluation of the hind limbs at week 4, week 8, and week 12 after the surgeries via using 1029-XL Incapacitance Tester (Columbus, Ohio, US). The formula for calculating the weight distribution ratio (%) of the injured leg was: [injured leg load (g)/uninjured leg load (g) + injured leg load (g)] × 100.
53. Perform H&E staining-based histopathology of rabbit major organs to verify whether there were any significant pathological abnormalities or degenerative changes, showing the implant’s *in vivo* biosafety.
54. Perform macroscopic observation and photography of the femur samples. The wound boundary visibility, filling of defect, regularity of joint surface and signs of inflammation were recorded. Meanwhile, perform ICRS macroscopic scoring evaluation (0–12) in terms of degree of defect repair (0–4), integration with border zone (0–4), and macroscopic appearance (0–4) (Supplementary Table S1) or/and Oswestry macroscopic scoring evaluation (0–10) in term of graft level with surrounding cartilage (0–2), integration with surrounding cartilage (0–2), appearance of surface (0–2), color of graft (0–2), and stiffness of the graft (0–2) (Supplementary Table S2).
55. Perform HF-ultrasound imaging on the medial femoral condyles using a linear array transducer (MS700, VevoLAZR, VisualSonics, Inc.) (Figures 5A–G) and IAUS scoring evaluation (0–4) (Supplementary Table S3). The ultrasound scanning parameters for 3D acquisition setup (e.g., scan distance: 14.021 mm; step size: 0.102 mm; scan frames: 138) were selected based on an early published study (Huang et al., 2018). The HF-ultrasound imaging could clearly distinguish the cartilage layer and cartilage-bone interface layer (Figure 5H).
56. A high-resolution micro-CT system (uCT40, Scanco Medical AG, Switzerland) was used to perform micro-CT scanning and 3D reconstruction on the medial femoral condyles to evaluate the quantity and quality of bone tissues (Figures 6A–F). The scanning parameters (e.g., the energy:70 kVp, 114 uA; high resolution; corresponding voxel size: 18 um; integration time: 200 ms) were selected. Within the VOI, several metric measures were calculated. These included the bone mineral density (BMD); the direct measurement of bone volume fraction (BV/TV), which is the ratio of bone volume over tissue volume; trabecular number (Tb.N); trabecular thickness (Tb.Th), which is the 3-D thickness; and trabecular separation (Tb.Sp). For more details, please refer to our previously published paper in Biomaterials (Lai et al., 2019).
57. Conduct the compression-based mechanical testing on the collected femur samples. Based on the typical load-displacement curves, reduced modulus (MPa) and hardness (MPa) of the regenerated tissues could be calculated (Li et al., 2023). If MMA-sectioned samples, micro-indentation-based mechanical testing was applied (Chow et al., 2014).
58. Conduct RT-qPCR or/and western blotting on biopsy-punched tissue samples. Alternatively, through bulk RNA-sequencing or/and proteomics, more valuable information might be obtained to analyze the molecular mechanisms of OCD repair. Before collecting tissue samples for testing, it is necessary to perform cardiac perfusion, which involves using saline or PBS to wash away the blood in the vessels, thus eliminating the influence of blood clotting on subsequent experiments. The specific operations are as follows: A large volume of sterilized saline or PBS must be perfused first, and after the tissue block is removed, it should be immersed into red blood cell lysis solution, followed by thorough washing with ice-cold PBS. It is recommended that RNA/protein extraction should be performed immediately after the sample collection. If immediate extraction is not possible, the sample should be flash-frozen in liquid nitrogen and stored at −80°C as soon as possible, and then RNA/protein can be extracted for subsequent analysis. This process ensures the integrity of the RNA and proteins and the quality of the results from further analyses. It’s crucial to handle the samples quickly and efficiently to prevent degradation
59. For histological staining analysis,
  (1) Specimens were decalcified by EDTA solution (0.5 M) for 1–2 months and the decalcification solution was replaced per week. The completion of sample decalcification was confirmed by x-ray radiography or needle stick without any obvious resistance (Figure 7).
  (2) Trim the samples to a suitable size until near the edge of the defects (Figure 7).
  (3) The decalcified specimens were dehydrated by immersion in 70%, 95%, and 100% ethanol for 1 day each. This step reduced the water content and preserved the tissue structure. After being cleared by incubation in xylene, these dehydrated samples were then infiltrated with melted paraffin and embedded (Figure 7).
  (4) A microtome (Leica RM2255, Leica, Germany) was used to perform a series of 5 um sections. The sectioning was standardized to collect the mid portion of the defect area for following histological staining analysis (Figure 7).
  (5) Then the section slides were used for various staining including hematoxylin and eosin (H&E) (Figure 8A), safranin O/fast green (SO&FG) (Figure 8B; Figure 8D), toluidine blue (T&B) (Figure 8C), Masson trichrome (M&T), Sirius red (S&R), and von Kossa staining.
  (6) Several scoring and validation systems were developed for quantification analysis, such as O’Driscoll histopathological classification system (Supplementary Table S4), the modified O’Driscoll histopathological classification system (Supplementary Table S5), ICRS-I histopathological classification system (Supplementary Table S6), and ICRS-II histopathological classification system (Supplementary Table S7).
  (7) For immunobiological (IHC) analysis, the expression of the following markers including Col I (Sigma C2456, 1:100), Col II (Neomarkers MS-235-P, 1: 100), Col X (Abcam, 1:150), VEGF (Santa Cruz sc-7269, 1:100), BMP2 (Abcam, ab6285, 1:1000), OPN (Abcam, 1:1000), OPG (Novus Biologicals, 1:100) TGF-β1 (Santa Cruz, 1:100), SOX9 (Abcam, 1:100), MMP13 (Proteintech, 1:100), and COX-2 (Santa Cruz c-1745, 1:100) was analyzed in the repaired area.
60. Statistical analysis.

The quantitative data from the implantation of PTM scaffolds as example implants and the Ctrl group were presented as mean ± standard deviations (SD). After the homogeneity test of variance, the Student’s t-test or the Mann-Whitney U, non-parametric test was used when comparing the two groups to determine statistical significance (*p* < 0.05). *p* values of statistical significances were represented as ∗∗∗*p* < 0.001, ∗∗*p* < 0.01, and ∗*p* < 0.05. The statistical analysis and graphing were performed using GraphPad Prism 9.0 (GraphPad Software Inc, CA, United States).

## 4 Anticipated results

### 4.1 Hematology test

**FIGURE 9:**
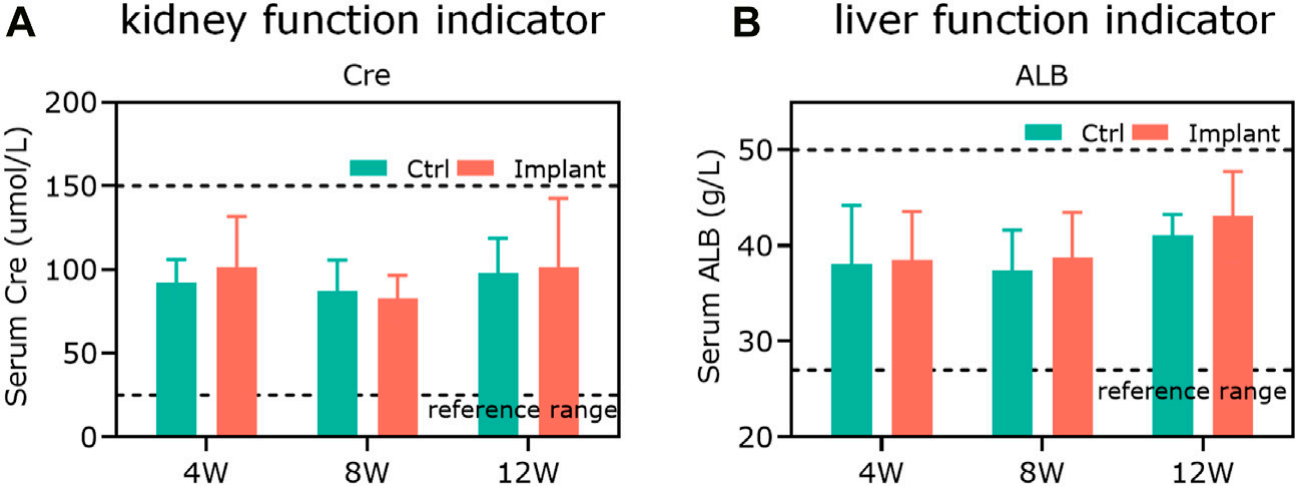
**T**he *in vitro* biosafety was confirmed by hematology tests. **(A)** The kidney function indicator (Cre) and **(B)** the liver function indicator (ALB) were within the safe and physiological ranges, showing that the scaffold implantation did not cause any obvious damage to the hematological system and the functions of rabbit livers and kidneys (*n* = 3).

The liver function indicators (ALB) and kidney function indicators (Cre) were within the safe and physiological regions (Figures 9A,B), indicating that the implantation of PTM scaffolds did not show any obvious damage to the hematological system and the functions of the liver and kidneys in a rabbit model.

### 4.2 Pain evaluation

**FIGURE 10:**
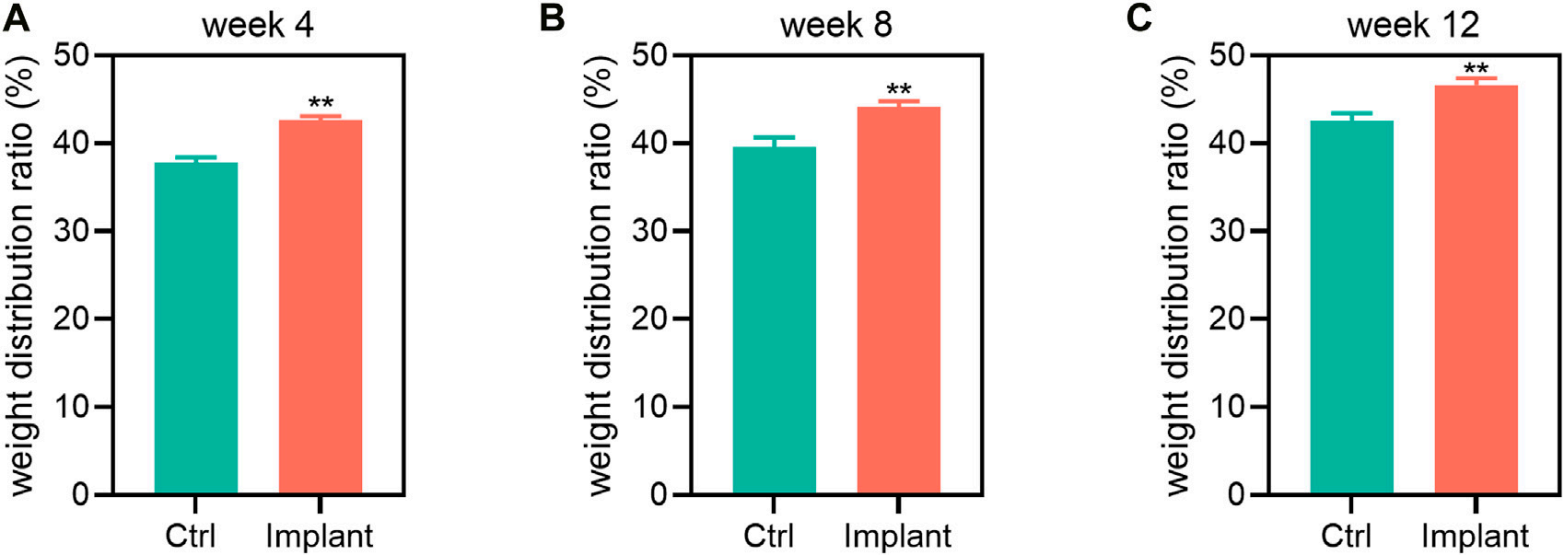
The pain assessment of the Ctrl and implant groups at **(A)** week 4, **(B)** week 8, and **(C)** week 12 post-implantation. The Mg-based scaffold implantation could notably elevate the weight distribution of the rabbit hind limbs, showing their pain alleviation effect (*n* = 6, Student’s t-test).

The pain measurement was conducted to see the weight distribution between the injured and uninjured hind legs post-operation, indicating how much pain the animals felt. We found that the scaffold implantation dramatically alleviated the pain compared to the control (Ctrl) group at all indicated time points (Figure 10). The pain alleviation effect might be largely contributed by the immunological and inflammatory regulation of the knee joint microenvironment via 3D implant-derived Mg ions (Yao et al., 2021).

### 4.3 *In vivo* biosafety test-staining of major organs

To evaluate the potential systemic side-effects of implants, the H&E staining-based histopathological analysis of the rabbit’s major internal organs (liver, heart, spleen, lung, and kidney) could be conducted. If the implanted scaffolds were excellent biocompatible, there would be no apparent abnormalities or degenerative changes in the Ctrl group and PTM group, suggesting the favorable biosafety and minimal systematic toxicity of implants.

### 4.4 Macroscopic assessment

**FIGURE 11:**
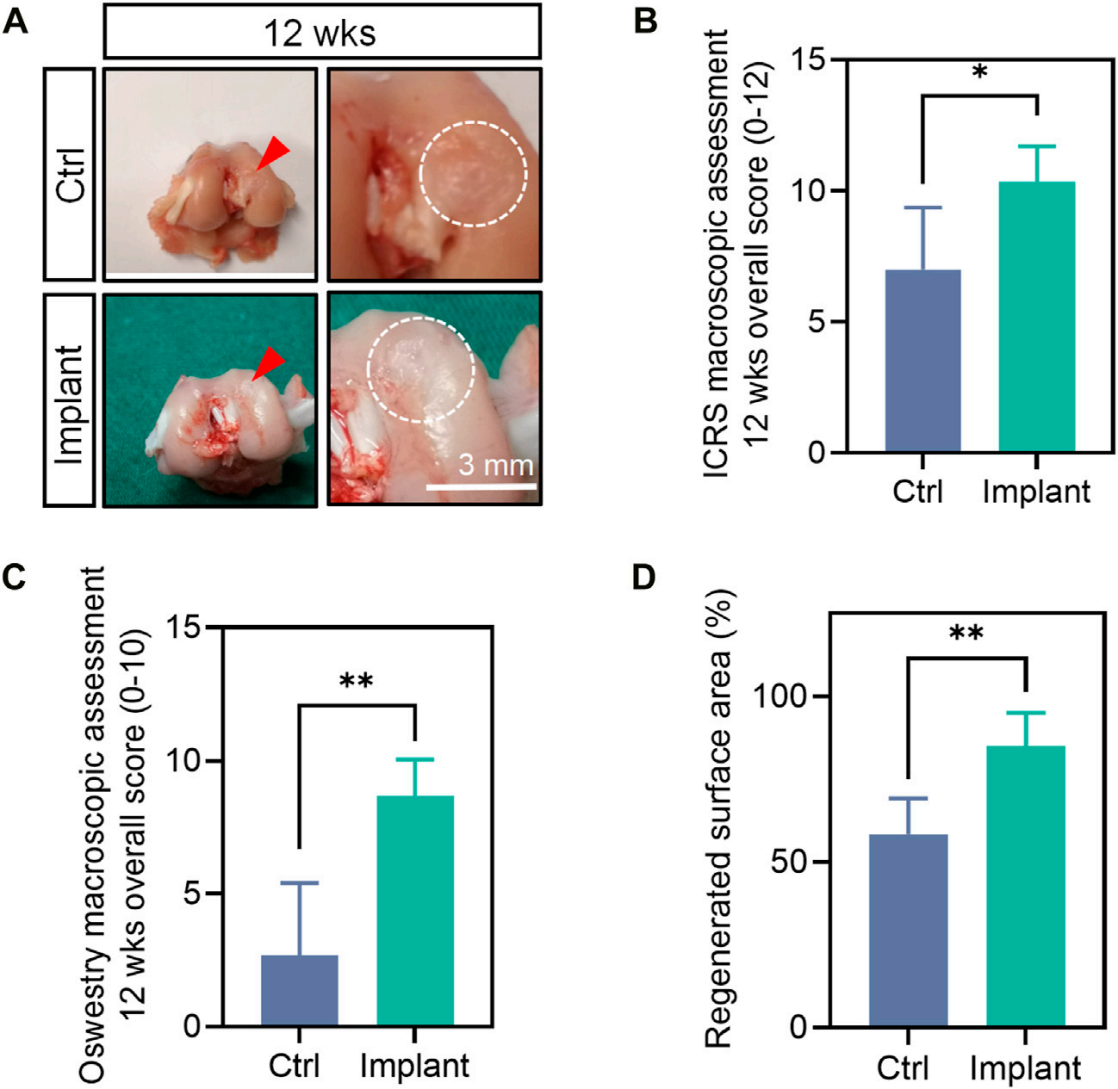
The gross appearance and macroscopic assessment of the harvested femur samples. **(A)** The gross appearance **(B)** ICRS macroscopic overall score, **(C)** Oswestry macroscopic overall score, **(D)** regenerated surface area of the femurs at 12 weeks post-surgically. (*n* = 6, the Mann-Whitney U, non-parametric test).

The gross appearance of the collected femur samples was recorded. We used the gross appearance of the femur samples (e.g., without the drainage of pus) as a preliminary indicator of the biocompatibility of our scaffolds. For the Ctrl group, the cartilage repair is insufficient and incomplete, with remaining holes or cracks, although they were irregularly covered with some newly-formed tissues to some extent. For the implants-treated group, more smooth and intact-looking cartilaginous tissues were regenerated within defects 12 weeks postoperation (Figure 11A). The quantification data showed that the regenerated surface area (%), ICRS macroscopic score and Oswestry macroscopic score of the implant group were markedly higher than the Ctrl group at week 12 (Figures 11B–D).

### 4.5 HF-ultrasound based evaluation

**FIGURE 12:**
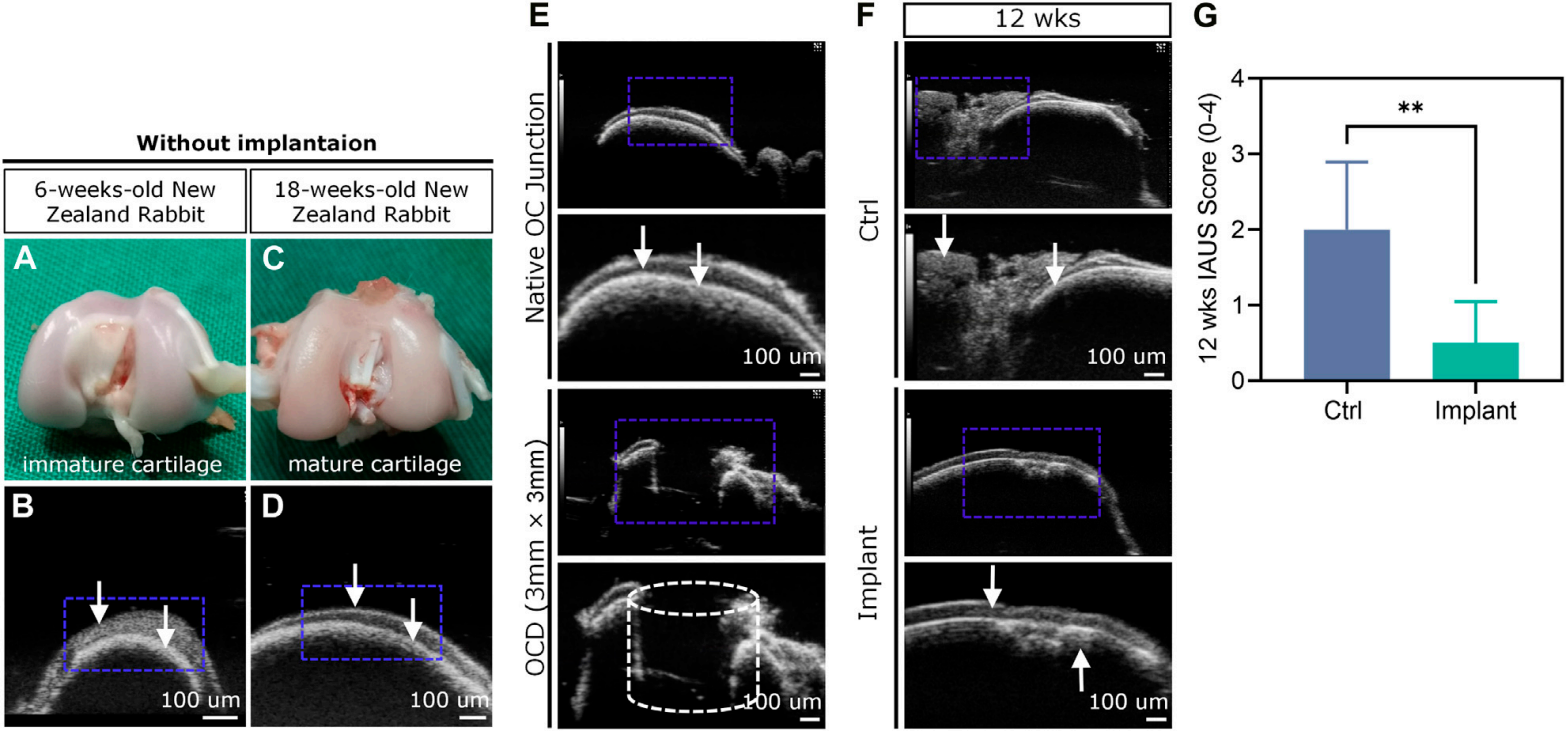
HF-ultrasound imaging-based assessment of OCD repair. **(A–D)** HF-ultrasound only can discriminate the structure of cartilage and cartilage-bone interface in an adult rabbit model. **(E)** HF-ultrasound can discriminate the healthy knee and OCD knee in an adult rabbit model. **(F)** The HF-ultrasound images and **(G)** IAUS score analysis of the repaired osteochondral tissues 12 weeks post-surgically (*n* = 6, Student’s t-test). The white arrows indicate the upper layer of cartilage and lower layer of bone-cartilage interface.

HF-ultrasound (≥20 MHz) scanning was incapable of clearly distinguishing the cartilage and cartilage-to-bone interface of young rabbits (i.e., 6-weeks-old) (Figures 12A,B), but this technique could achieve this goal in older rabbits (i.e. 18-weeks-old) (Figures 12C,D) and clearly detect a 3 mm × 3 mm OCD (Figure 12E). For the Ctrl group, the defect could not heal well spontaneously and was accompanied by uneven surfaces, cracks, and unfilled areas, while the implant-treated group had a smoother surface and regenerated cartilage-to-bone interface (Figure 12F). The IAUS score showed that the scaffold implantation could notably improve osteochondral regeneration (Figure 12G).

### 4.6 Micro-CT assessment

**FIGURE 13:**
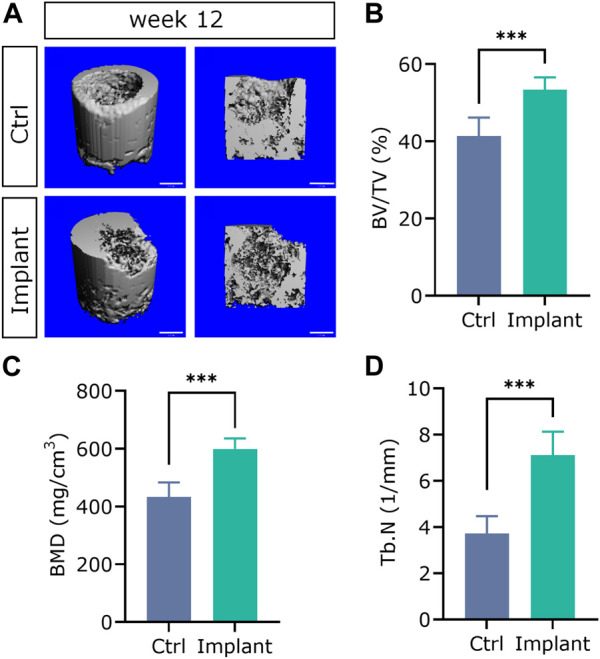
The 3D reconstructed micro-CT images **(A)** and quantification analysis of subchondral bone regeneration, including **(B)** BV/TV (%), **(C)** BMD, and **(D)** Tb.N. (*n* = 6, Student’s t-test; scale bar = 1 mm).

The representative 3D reconstructed images of mineralized tissues are shown in Figure 13A. And the quantification data of BV/TV(%), BMD, and Tb.N in the implant group were significantly higher than that in the Ctrl group, suggesting that the enhanced subchondral bone regeneration effect was achieved by the favorable scaffolds implantation (Figures 13B–D). These findings were in accordance with our previously published paper in a rabbit SAON (steroid-associated osteonecrosis) model (Lai et al., 2019).

### 4.7 Histology and histomorphometry

**FIGURE 14:**
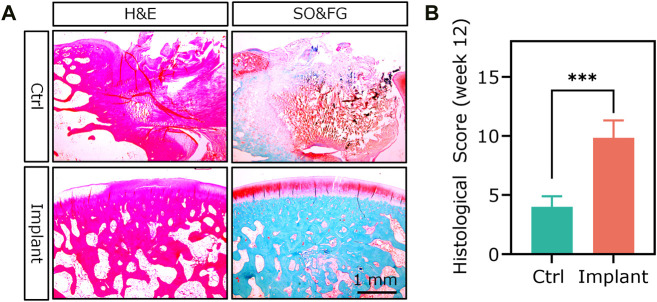
The H&E staining and SO&FG staining **(A)** and **(B)** ICRS histological scoring analysis of the repaired osteochondral tissues at week 12. (*n* = 6, Student’s t-test).

Histological analysis was the most broadly used method for assessing the degree of OCD repair as well as the components and distribution of neo-tissues. The representative section staining images of H&E and SO&FG are shown in Figure 14A. H&E staining could provide the general morphology of repaired osteochondral tissues. Hematoxylin stains the nuclei blue to dark, while eosin stains the extracellular matrix (ECM) and cytoplasm red to pink. For the Ctrl group, the repaired cartilage was obviously uneven and thinner; while in the implant group, the repaired cartilage was smoother, and more complete, and the repaired subchondral bone area was denser (Figure 14A). SO&FG staining was widely used to stain acidic proteoglycan in cartilage. The cartilage was stained orange to red; the nuclei were stained black, and the background was stained green. The proteoglycan contents in the implant group were more than that in the Ctrl group (Figure 14A). Additionally, T&B staining was always used to visualize GAG contents. M&T and S&R staining was conducted to visualize collagen fibers. von Kossa staining was utilized to visualize calcium contents. The selection of which staining method depends on the study purposes and implants. The ICRS histological score at week 12 showed that PTM implants could remarkably augment the dual-lineage osteochondral regeneration (Figure 14B). IHC can help to assess the quantity and quality of ECM components, such as glycosaminoglycans, proteoglycans, and collagen, that are essential for the structure and function of osteochondral unit. In the scenario of OCD repair, IHC could be a powerful approach for distinguishing whether the regenerated cartilage was hyaline (Col II), fibrous (Col I), or hypertrophic (Col X). Additionally, IHC can assist in identifying the type and origin of the cells that are involved in the repairing process. For the histomorphometry analysis of stained sections, various scoring and validation systems were developed for quantification.

### 4.8 Mechanical testing

The biomechanical properties of the reparative cartilage were assessed by mechanical testing. For example, the nano/micro-indentation experiments could provide reduced modulus and hardness (Chow et al., 2014). Theoretically, these two indicators could be significantly elevated by the favorable scaffold-based OCD repair.

### 4.9 RT-qPCR

Usually, the RT-qPCR analysis of the biopsy-punched tissue samples could reveal the expression of a panel of related genes in the repaired osteochondral unit.

### 4.10 Bulk RNA-sequencing and proteomics

These two methods can provide valuable information about the biological processes and pathways involved in osteochondral repair, such as cell differentiation, ECM synthesis, inflammation, angiogenesis, and signaling crosstalk. However, bulk RNA sequencing and proteomics also have some limitations and challenges. For example, bulk RNA sequencing cannot capture the heterogeneity and spatial distribution of different cell types within a tissue, while proteomics cannot identify the exact origin and function of post-translational modifications. Therefore, combining bulk RNA sequencing and proteomics with other techniques, such as single-cell analysis, imaging, and bioinformatics, can enhance the understanding of osteochondral repair at multiple levels.

## 5 Discussion

Tissue-engineered implants are promising therapies for healing OCD; however, well-designed animal studies are still the gold standard before their translation into clinical practice despite U.S. FDA no longer requires animal tests before human drug trials since December 2022 (Han, 2023). This paper intends to provide a rabbit OCD model for preliminary proof-of-concept studies and recommend a series of subsequent assessment methodologies to investigate the implant’s *in vivo* biosafety and repairing efficacy. Although rabbit models have several strengths, compared with rat, mouse, dog, mini-pig, ship, goat, horse, and monkey, rabbit has some disadvantages and limitations in studying OCD repair. Rabbits reach skeletal maturity at around 9 months, which can limit the window for studying long-term osteochondral repair processes (Chu et al., 2010; Cook et al., 2014; Meng et al., 2020). Concerning joint size and cartilage thickness, while larger and thicker than rodents, rabbit joints and cartilage are smaller and thinner than those of lager animals and humans, which may not accurately represent human osteochondral pathology and affect the translatability of preclinical results (Chu et al., 2010; Cook et al., 2014; Meng et al., 2020). What’s more, the biomechanical properties of rabbit joints (e.g., weight-bearing and mechanical load) significantly differ from those of humans, which also can impact the applicability of findings to human joint repair (Chu et al., 2010; Cook et al., 2014; Meng et al., 2020). More importantly, rabbits have a faster bone growth rate and higher regenerative capacity than humans, which might lead to overestimation of the efficacy of proposed repair strategies (Chu et al., 2010; Cook et al., 2014; Meng et al., 2020). The use of 4-month-old rabbits, whose growth plates have not yet closed, presents a limitation as it may not accurately represent OCD repair in mature individuals. The growth plates’ status can influence the healing process, and using animals at this stage of development could lead to results that are not fully applicable to adult populations. These factors usually impact the translatability of research findings from rabbits to humans, and the results need to be validated in larger animal models that are more anatomically and biomechanically similar to humans before clinical translation.

Additionally, for the implantation of PTM scaffolds as an example for OCD repair, while 6 rabbits per group may be sufficient for preliminary studies to identify improvements in healing, larger sample sizes are generally required for comparing multiple implant groups (e.g., scaffolds +/− Mg). This is to ensure statistical power and to account for biological variability. The absence of bioinformatic-based analysis such as RNA-seq or proteomics is a missed opportunity for unbiased, comprehensive evaluation of the repair process at the molecular level. Incorporating such methods could provide insights into the pathways involved in healing and the biological mechanisms by which different treatments exert their effects. Longer-term studies (e.g., 6 months and 12 months) are also highly needed to assess the durability and functionality of the repaired tissue over time.

To date, we usually cannot directly compare different preclinical animal testing regarding OCD regeneration, mainly due to the lack of standardized disease models and evaluation approaches. Wide varieties of animal models including rabbits, study durations (2–48 weeks), defect models (acute vs. chronic), defect locations (groove vs. loading bearing area), defect sizes (3–5 mm), control groups, histological/radiological tests, and semi-quantification/scoring systems were reported. Unfortunately, no widely acknowledged consensus on the above problems exists currently. The streamlined and standardized animal models and evaluation methods could promote the predictive accuracy, reproducibility, and regulatory approval of the translation research of innovative implants for healing OCD. It is envisioned that more preclinical studies will be conducted based on a more standardized rabbit OCD model with consistent subsequent assessments, to better compare the results between labs and facilitate the progress from bench side to bed.

## 6 Conclusion

In this article, we presented an osteochondral study protocol including the establishment of a rabbit OCD model and a series of subsequent assessment methodologies to investigate the implant’s *in vivo* biosafety and repairing efficacy. Using this model, many scaffold biomaterials and tissue-engineered grafts could be implanted, and their *in vivo* biosafety could be evaluated by hematology test and H&E staining-based histopathology of major inner organs as well as the healing ability could be compared via macroscopic appearance, pain measurement, HF-ultrasound, micro-CT, mechanical testing, histological and histomorphometric analysis, RT-qPCR, western blotting, bulk RNA-sequencing, and proteomics. This well-established rabbit OCD model will expedite the validation and translation of novel implants into clinical settings. Next-generation therapeutics will largely profit from reliable and easy-to-use *in vivo* models.

## 7 Statement of significance

Given the regulatory requests to assess the *in vivo* biosafety and efficacy of osteochondral implants, we here present a reliable and standardized rabbit OCD model for objective evaluation in osteochondral tissue engineering. This model can be used to evaluate the therapeutic effects of different interventions, including a variety of 1osteochondral grafting scaffolds and tissue-engineered (bio) products that aim to repair OCD and restore joint function. It will expedite the validation and translation of novel implants into clinical settings. Innovative treatments will be easily confirmed experimentally by using this established *in vivo* model.

## Data Availability

The original contributions presented in the study are included in the article/Supplementary Material, further inquiries can be directed to the corresponding authors.

## Appendix

The relevant animal experimentation ethics (Ref. No. 17-172-AOF) is attached in the Appendix.
