# Supplementary material for: A rabbit osteochondral defect (OCD) model for evaluation of tissue engineered implants on their biosafety and efficacy in osteochondral repair
Source: Front Bioeng Biotechnol. 2024 May 3;12:1352023. doi: 10.3389/fbioe.2024.1352023 (PMC11099227; doi:10.3389/fbioe.2024.1352023)
Supplement: Supplementary file 1 [file Table1.docx]

**Supplementary Materials**

Title: A Rabbit Osteochondral Defect (OCD) Model for Evaluation of Tissue Engineered Implants on their Biosafety and Efficacy in Osteochondral Repair

Liangbin Zhou ^1,2^, Kevin Ki-Wai Ho ^1,***^, Zhenli Zheng ^1^, Jiankun Xu ^1^, Ziyi Chen ^1^, Xiangdong Ye ^3^, Li Zou ^1,4^, Ye Li ^1^, Liang Chang ^1^, Hongwei Shao ^1^, Xisheng Li ^5,6^, Jing Long ^7^, Yangyi Nie ^7^, Martin J. Stoddart ^8^, Yuxiao Lai ^7,**^, Ling Qin ^1,7,*^

Correspondence: [kevinho@cuhk.edu.hk](mailto:kevinho@cuhk.edu.hk); [yx.lai@siat.ac.cn](mailto:yx.lai@siat.ac.cn); and [lingqin@cuhk.edu.hk](mailto:lingqin@cuhk.edu.hk);

**Table S1. Macroscopic classification systems according to Outerbridge (Outerbridge, 1961) and ICRS-CRA (Cartilage Repair Assessment)**

| **Classification**  **system** | **Item** | **Score** | **Description** |
| --- | --- | --- | --- |
| **Outerbridge** |  | 0 | Normal cartilage |
|  |  | 1 | Cartilage with softening and swelling |
|  |  | 2 | A partial-thickness defect with fissures on the surface that do not reach subchondral bone  or exceed 1.5 cm in diameter |
|  |  | 3 | Fissuring to the level of subchondral bone in an area with a diameter more than 1.5 cm |
|  |  | 4 | Exposed subchondral bone |
| **ICRS-CRA** | **Degree of defect repair** | 4 | In level with surrounding cartilage |
|  |  | 3 | 75 % repair of defect depth |
|  |  | 2 | 50 % repair of defect depth |
|  |  | 1 | 25 % repair of defect depth |
|  |  | 0 | 0 % repair of defect depth |
|  | **Integration to border zone** | 4 | Complete integration with surrounding cartilage, Demarcating border < 1 mm |
|  |  | 3 | 75 % of graft integrated, 25 % with notable border > 1 mm width |
|  |  | 1 | 50 % of graft integrated with surrounding cartilage, 50 % with a notable border > 1 mm |
|  |  | 0 | From no contact to 25 % of graft integrated with surrounding cartilage |
|  | **Macroscopic appearance** | 4 | Intact smooth surface |
|  |  | 3 | Fibrillated surface |
|  |  | 2 | Small, scattered fissures or cracks |
|  |  | 1 | Several, small or few but large fissures |
|  |  | 0 | Total degeneration of grafted area |
|  | **Maximal cumulative score** | 12 |  |

**Table S2.** **Macroscopic classification systems according to Oswestry score (Smith et al., 2005)**


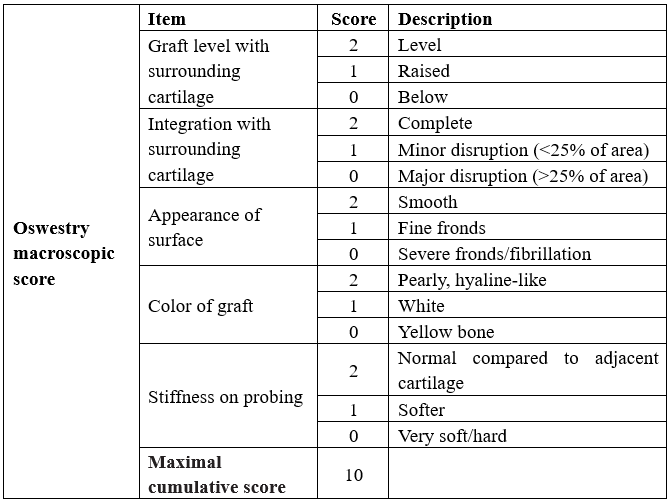


**Table S3.** **The intra-articular ultrasound score (IAUS) for the evaluation of cartilage (Kaleva et al., 2011)**

| **Grade** | **Description of the conditions of article cartilage tissue** |
| --- | --- |
| 0 | Normal |
| 1 | Nearly normal. Superficial lesions. Superficial fissures and cracks. |
| 2 | Abnormal. Lesions extending down to lower than fifty percent of the cartilage depth. |
| 3 | Severely abnormal. Cartridge defects extending down larger than fifty percent of the cartilage depth as well as down to the calcified layer and down to but not through the subchondral bone. Blisters are induced in this grade. |
| 4 | Severely abnormal. Cartilage defects extending through the subchondral bone. |

**Table S4. Histopathological classification systems according to O’Driscoll (O’Driscoll et al., 1998)**

| **Item** | **Score** | **Description** |
| --- | --- | --- |
| 1 – Cellular morphology | 4 | Hyaline articular cartilage |
|  | 2 | Incomplete differentiated mesenchyme |
|  | 0 | Fibrous tissue or bone |
| 2 – Safranin-O staining of the matrix | 3 | Normal or nearly normal |
|  | 2 | Moderate |
|  | 1 | Slight |
|  | 0 | None |
| 3 – Surface regularity | 3 | Smooth and intact |
|  | 2 | Superficial horizontal lamination |
|  | 1 | Fissures – 25-100 % of the thickness |
|  | 0 | Severe disruption, including fibrillation |
| 4 – Structural integrity | 2 | Normal |
|  | 1 | Slight disruption, including cysts |
|  | 0 | Severe disintegration |
| 5 – Thickness | 2 | 100 % of normal adjacent cartilage |
|  | 1 | 50-100 % of normal cartilage |
|  | 0 | 0-50 % of normal cartilage |
| 6 – Bonding to adjacent cartilage | 2 | Bonded at both ends of graft |
|  | 1 | Bonded at one end, or partially at both ends |
|  | 0 | Not bonded |
| 7 – Hypocellularity | 3 | Normal cellularity |
|  | 2 | Slight hypocellularity |
|  | 1 | Moderate hypocellularity |
|  | 0 | Severe hypocellularity |
| 8 – Chondrocyte clustering | 2 | No clusters |
|  | 1 | < 25 % of the cells |
|  | 0 | 25-100 % of the cells |
| 9 – Freedom from degenerative changes in adjacent cartilage | 3 | Normal cellularity, no clusters, normal staining |
|  | 2 | Normal cellularity, mild clusters, moderate staining |
|  | 1 | Mild or moderate hypocellularity, slight staining |
|  | 0 | Severe hypocellularity, poor or no staining |
| **Maximal cumulative score** | 24 |  |

**Table S5. The modified O’Driscoll scoring scale for the evaluation of (a) cartilage and (b) subchondral bone repair in osteochondral tissue engineering (Cheuk et al., 2011)**


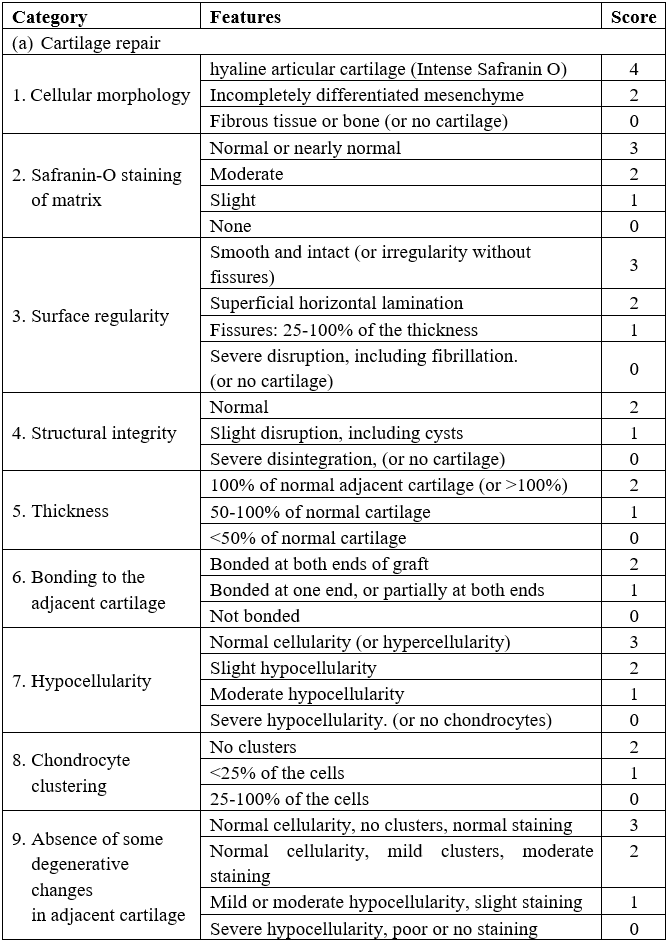


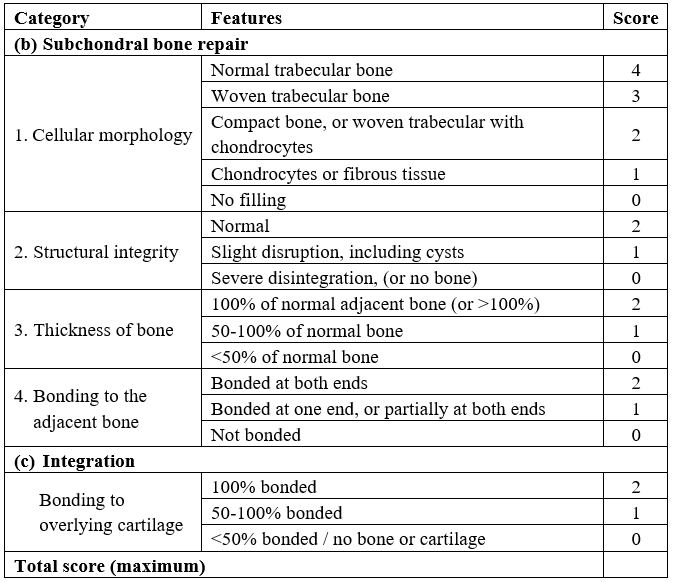


**Table S6. ICRS-I histopathological classification systems (Mainil-Varlet *et al*., 2003)**

| **Item** | **Score** | **Description** |
| --- | --- | --- |
| I – Surface | 3 | Smooth/continuous |
|  | 0 | Discontinuities/irregularities |
| II – Matrix | 3 | Hyaline |
|  | 2 | Mixture: hyaline/fibrocartilage |
|  | 1 | Fibrocartilage |
|  | 0 | Fibrous tissue |
| III – Cell distribution | 3 | Columnar |
|  | 2 | Mixed/columnar-clusters |
|  | 1 | Cluster |
|  | 0 | Individual cells/disorganized |
| IV – Cell population viability | 3 | Predominantly viable |
|  | 1 | Partially viable |
|  | 0 | < 10 % viable |
| V – Subchondral bone | 3 | Normal |
|  | 2 | Increased remodeling |
|  | 1 | Bone necrosis/granulation tissue |
|  | 0 | Detached/fracture/callus at base |
| VI – Cartilage mineralization (calcified cartilage) | 3 | Normal |
|  | 0 | Abnormal/inappropriate location |
| **Maximal cumulative score** | 18 |  |

**Table S7. ICRS-II histopathological classification systems (Mainil-Varlet et al., 2010)**

| **Item** | **Score** | **From 0** | **to** | **100** |
| --- | --- | --- | --- | --- |
| 1 – Tissue morphology (viewed under polarized light) | 0-100 | Full-thickness collagen fibers |  | Normal cartilage birefringence |
| 2 – Matrix straining (metachromasia) | 0-100 | No staining |  | Full metachromasia |
| 3 – Cell morphology | 0-100 | No round/oval cells |  | Mostly round/oval cells |
| 4 – Chondrocyte clustering (4 or more grouped cells) | 0-100 | Present |  | Absent |
| 5 – Surface architecture | 0-100 | Delamination, or major irregularity |  | Smooth surface |
| 6 – Basal integration | 0-100 | No integration |  | Complete integration |
| 7 – Formation of a tidemark | 0-100 | No calcification front |  | Tidemark |
| 8 – Subchondral bone abnormalities/marrow fibrosis | 0-100 | Abnormal |  | Normal marrow |
| 9 – Inflammation | 0-100 | Present |  | Absent |
| 10 – Abnormal calcification/ossification | 0-100 | Present |  | Absent |
| 11 – Vascularization (within the repair tissue) | 0-100 | Present |  | Absent |
| 12 – Surface/superficial assessment | 0-100 | Total loss or complete disruption |  | Resembles intact articular cartilage |
| 13 – Mid/deep zone assessment | 0-100 | Fibrous tissue |  | Normal hyaline cartilage |
| 14 – Overall assessment | 0-100 | Bad (fibrous tissue) |  | Good (hyaline cartilage) |
| **Cumulative score (mean)** | 0-100 |  | | |
